# Supplementary material for: Large differences in regional precipitation change between a first and second 2 K of global warming
Source: Nat Commun. 2016 Dec 6;7:13667. doi: 10.1038/ncomms13667 (PMC5150659; doi:10.1038/ncomms13667)
Supplement: Supplementary Information — Supplementary Figures 1-4, Supplementary Table 1, Supplementary Notes 1-2 and Supplementary References. [file ncomms13667-s1.pdf]

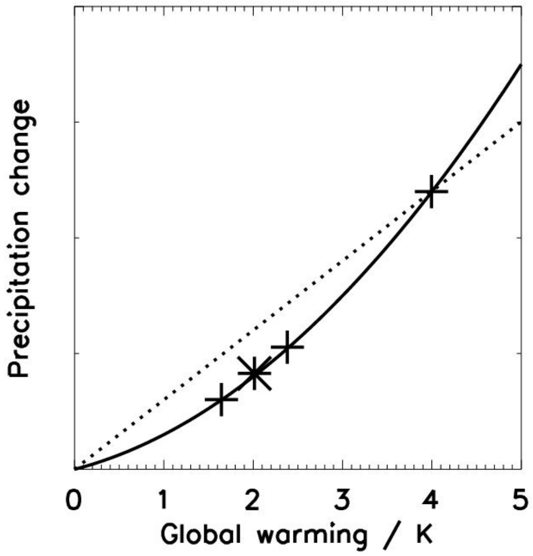

Supplementary Figure 1. Illustrating how nonlinearity might affect the averaged mitigation scenario. Precipitation change is plotted against global warming, for an idealized purely nonlinear response (solid curve; dotted line illustrates an approximation that precipitation is proportional to global warming). Crosses represent rcp2.6, rcp4.5 and rcp8.5. Asterisk: the mean of rcp2.6 and rcp4.5 (representing our averaged mitigation scenario).

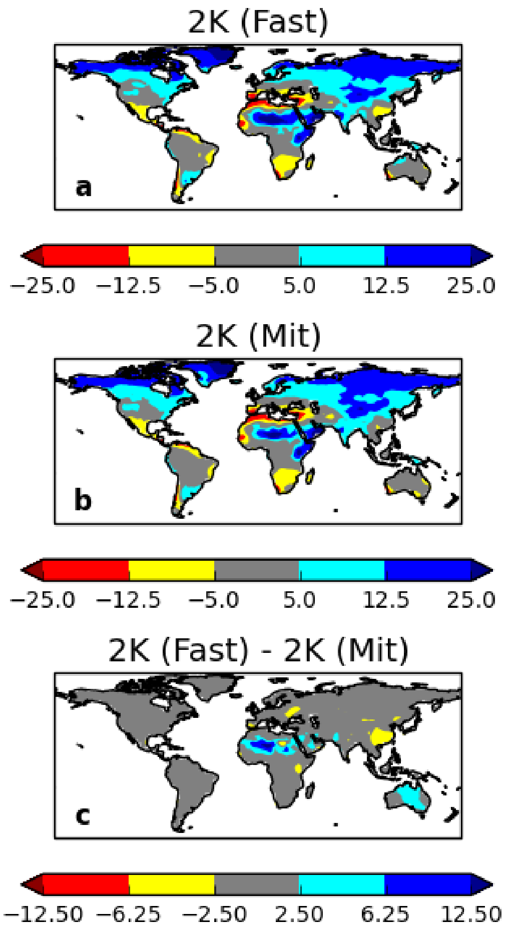

Supplementary Figure 2. Ensemble mean precipitation differences. As Figure 2a-c, but precipitation changes are converted to percentages of the pre-industrial climate mean for each model individually, before ensemble means are taken. Results are shown for change along **a** the fast (high-forcing) and **b** the mitigation routes to a first 2K; and **c**, their difference (scale for panel **c** is half that of **a**,**b**). The differences over the Sahara in panel **c** are exaggerated here due to the very low climatological precipitation in this region.

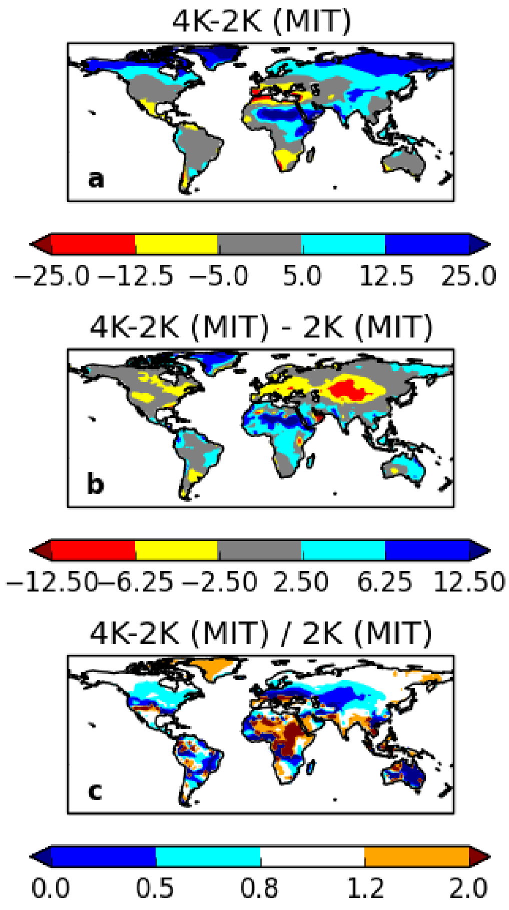

Supplementary Figure 3. Ensemble mean precipitation differences. As Figure 2d-f, but with precipitation changes expressed as percentages of the pre-industrial means for each model, before ensemble means are taken. Results are shown **a** for differences for the second 2K; **b**, the second 2K minus the first 2K under mitigation conditions (note different scale). **c**: The second 2K divided by the first 2K under mitigation conditions. Results for the 1pctCO2 experiment are not included because of the strong similarity between Supplementary Figure 3c, and Figure 2f (these ratios are insensitive to whether precipitation is in mm/day or %).

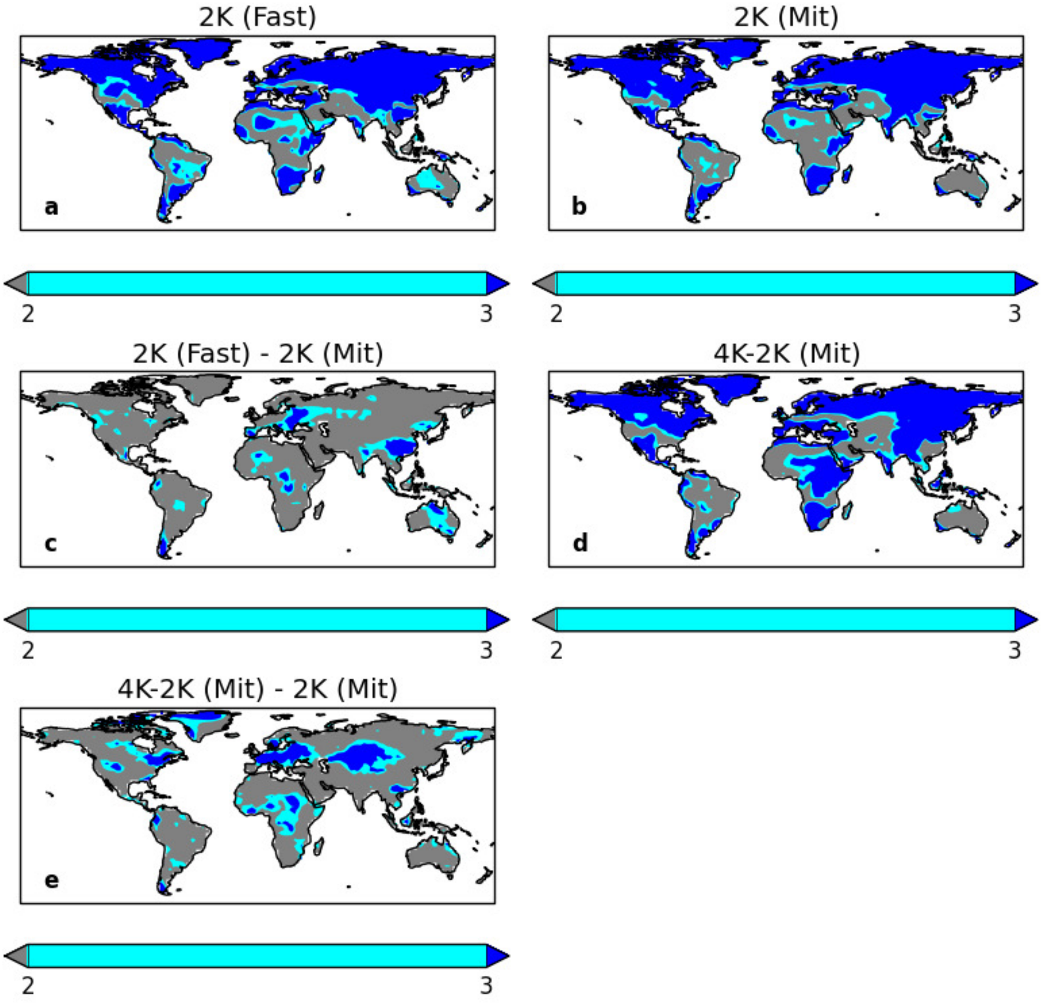

Supplementary Figure 4. Signal/noise ratio in the ensemble mean results of Figure 2, including both model spread and internal variability in the 'noise'. As Figure 2a-e, but showing, in each panel, the ratio: |ensemble mean| / (standard deviation in the ensemble mean). The standard deviation in the ensemble mean is estimated simply by dividing the ensemble standard deviation by $n^{1/2}$, where n is the number of models. Results are shown for change along **a** the fast (high-forcing) and **b** the mitigation routes to a first 2K; and **c**, their difference (scale for panel **c** is half that of **a**,**b**). **d** shows differences for the second 2K; **e**, the second 2K minus the first 2K under mitigation conditions.

Modeling Center (or Group)	Institute ID	Model Name
Beijing Climate Center, China Meteorological Administration	BCC	BCC-CSM1.1
College of Global Change and Earth System Science, Beijing Normal University	GCESS	BNU-ESM
Canadian Centre for Climate Modelling and Analysis	CCCMA	**CanESM2**
National Center for Atmospheric Research	NCAR	**CCSM4**
Community Earth System Model Contributors	NSF-DOE-NCAR	CESM1(CAM5)
Centre National de Recherches Météorologiques / Centre Européen de Recherche et Formation Avancée en Calcul Scientifique	CNRM-CERFACS	**CNRM-CM5**
Centre National de Recherches Météorologiques / Centre Européen de Recherche et Formation Avancée en Calcul Scientifique Commonwealth Scientific and Industrial Research Organization in collaboration with Queensland Climate Change Centre of Excellence	CNRM-CERFACS CSIRO-QCCCE	**CSIRO-Mk3.6.0**
LASG, Institute of Atmospheric Physics, Chinese Academy of Sciences and CESS,Tsinghua University	LASG-CESS	**FGOALS-g2**
LASG, Institute of Atmospheric Physics, Chinese Academy of Sciences	LASG-IAP	FGOALS-gl
The First Institute of Oceanography, SOA, China	FIO	FIO-ESM
NOAA Geophysical Fluid Dynamics Laboratory	NOAA GFDL	**GFDL-CM3**<br>**GFDL-ESM2G**<br>**GFDL-ESM2M**
NASA Goddard Institute for Space Studies	NASA GISS	GISS-E2-H (p1)<br>GISS-E2-R (p1)
National Institute of Meteorological Research/Korea Meteorological Administration.<br>Met Office Hadley Centre.	NIMR/KMA MOHC	**HadGEM2-AO**
Institut Pierre-Simon Laplace	IPSL	**IPSL-CM5A-LR**<br>**IPSL-CM5A-MR**
Japan Agency for Marine-Earth Science and Technology, Atmosphere and Ocean Research Institute (The University of Tokyo), and National Institute for Environmental Studies	MIROC	**MIROC-ESM**<br>MIROC-ESM-CHEM

Max-Planck-Institut für Meteorologie (Max Planck Institute for Meteorology)	MPI-M	**MPI-ESM-MR**<br>**MPI-ESM-LR**
Meteorological Research Institute	MRI	**MRI-CGCM3**
Norwegian Climate Centre	NCC	**NorESM1-M**<br>NorESM1-ME

Supplementary Table 1. List of CMIP5 models used. The subset of models used in Figure 1 are highlighted in bold.

Supplementary Note 1 - the use of an averaged mitigation scenario

As stated in the main text, an averaged mitigation scenario (the mean of rcp2.6 and rcp4.5 at the end of the century) is used. Here, the benefits and potential issues of this approach are discussed.

There are two reasons for averaging rcp2.6 and rcp4.5. First, this averaged scenario has an ensemble mean, global mean temperature very close to 2K above pre-industrial levels. This permits us to compare climate change across different intervals with the same ensemble mean global warming (2K). Second, the mean reduces the influence of internal variability (giving a 60-year mean).

We discuss potential issues with this approach in terms of linear and non-linear mechanisms, and responses to different forcings.

By definition, there is no issue with averaging linear mechanisms across two scenarios: if all mechanisms were linear, the resulting mean would correspond to a scenario with forcing history mid-way between rcp2.6 and rcp4.5. This is the basis of response-function models[1].

Non-linear responses are also unlikely to be an issue, because rcp2.6 and rcp4.5 are relatively close in terms of global mean warming by the end of the century (they are separated by less than 1K). This is illustrated in Supplementary Figure 1, which

shows an idealized precipitation response with relatively large non-linearity: the dotted line is not a good approximation of the solid curve. However, the mean of rcp2.6 and rcp4.5 (our averaged mitigation scenario) lies very close to the solid curve, because these two scenarios are close in global mean warming.

Averaging rcp2.6 and rcp4.5 will, however, remove some scenario-specific effects of non-greenhouse gas forcings, such as land-use change. Therefore, the influence of non-greenhouse gas forcings in causing different precipitation patterns may be under-estimated in our analysis.

Supplementary Note 2 - the role of fast responses to radiative forcing in Figure 2c

As stated in the main text: away from China, the small differences in Figure 2c are broadly consistent with linear mechanisms associated with fast responses to radiative forcing. This is discussed below.

Regional precipitation responds to radiative forcing change over different timescales[2,3]. Fast responses are partly driven by rapid land surface warming (associated with minimal global-mean warming), while slower responses are limited by timescales of ocean warming (similar timescales to global-mean warming). Two scenarios with the same global-mean temperature (and so, similar slow precipitation responses) may have different regional precipitation patterns if their radiative forcing differs (causing different fast precipitation responses).

The two 2K intervals compared in Figure 2c have the same global mean temperature, but a global-mean radiative forcing difference of about 0.5 W m^{-2} (see Figure 1; this arises because forcing is increasing more rapidly in the rcp8.5 scenario, and because of the lagged response of global warming to forcing change). Therefore, differences in Figure 2c are expected, in part from this 0.5 W m^{-2} difference in forcing (via fast precipitation responses). The ensemble mean fast precipitation response to a quadrupling of CO_2 (corresponding[4] to an ensemble mean radiative forcing change of about 7 W m^{-2}) has been estimated from experiments where forcings are changed with the sea-surface temperatures held constant[2]. This is shown in Figure 2 of Richardson

et al. (2015). The regional extrema in this pattern correspond in sign and location to the small features in Figure 2c (away from China). Scaling their figure by the ratio 0.5/7 (and converting from units of mm/year to mm/day), implies that values of around 250 mm/year in Richardson et al. (2015) would translate to 0.05 mm/day in Figure 2c of the current manuscript. This is consistent with only small localized effects being visible in Figure 2c of the current manuscript.

Supplementary References

1 Good, P., Gregory, J. M. & Lowe, J. A. A step-response simple climate model to reconstruct and interpret AOGCM projections. *Geophysical Research Letters* **38**, -, doi:Artn L01703
Doi 10.1029/2010gl045208 (2011).

2 Richardson, T. B., Forster, P. M., Andrews, T. & Parker, D. J. Understanding the Rapid Precipitation Response to CO2 and Aerosol Forcing on a Regional Scale. *Journal of Climate* **29**, 583-594 (2016).

3 Chadwick, R., Good, P., Andrews, T. & Martin, G. Surface warming patterns drive tropical rainfall pattern responses to CO2 forcing on all timescales. *Geophysical Research Letters* **41**, 610-615, doi:10.1002/2013GL058504 (2014).

4 Andrews, T., Gregory, J. M., Webb, M. J. & Taylor, K. E. Forcing, feedbacks and climate sensitivity in CMIP5 coupled atmosphere-ocean climate models. *Geophysical Research Letters* **39**, doi:Artn L09712
Doi 10.1029/2012gl051607 (2012).
